# Testosterone attenuates pulmonary epithelial inflammation in male rats of COPD model through preventing NRF1-derived NF-κB signaling

**DOI:** 10.1093/jmcb/mjaa079

**Published:** 2021-01-20

**Authors:** Xueting Wang, Linlin Huang, Shan Jiang, Kang Cheng, Dan Wang, Qianqian Luo, Xiaomei Wu, Li Zhu

**Affiliations:** Institute of Special Environmental Medicine, Nantong University, Nantong 226019, China

**Keywords:** testosterone replacement therapy, COPD, NRF1, NF-κB, transcriptional regulation, endothelia

## Abstract

Testosterone deficiency is common in male patients with chronic obstructive pulmonary disease (COPD) and may correlate with the deterioration of COPD. Clinical research suggests that testosterone replacement therapy may slow the COPD progression, but the specific biological pathway remains unclear. In this study, we explored the effect of testosterone on pulmonary inflammation in male COPD rats. The animals were co-treated with lipopolysaccharide (LPS) and cigarette to induce COPD. In COPD rats, nuclear respiratory factor 1 (NRF1) and NF-κB p65 were upregulated. In cigarette smoke extract (CSE)-, LPS-, or the combination of CSE and LPS-treated L132 cells, NRF1 and p65 were also upregulated. Silencing NRF1 resulted in the downregulation of p65. ChIP‒seq, ChIP‒qPCR, and luciferase results showed that NRF1 transcriptionally regulated p65. Both male and female COPD rats showed an upregulated NRF1 level and similar pulmonary morphology. But NRF1 was further upregulated in male castrated rats. Further supplementing testosterone in castrated male rats significantly reduced NRF1, pulmonary lesions, and inflammation. Supplementation of testosterone also reduced the phosphorylation of p65 and IKKβ induced by LPS or CSE in L132 cells. Our results suggest that testosterone plays a protective role in pulmonary epithelial inflammation of COPD through inhibition of NRF1-derived NF-κB signaling and the phosphorylation of p65.

## Introduction

Chronic obstructive pulmonary disease (COPD) is the third leading cause of non-communicable diseases and brings major healthcare burden worldwide (World Health Organization, 2018). The prevalence is higher in men than women (Wang et al., 2018). Men with COPD often have hypoxemia, systemic inflammation, and multiple comorbidities, increasing the risk of hypogonadism (Balasubramanian and Naing, 2012). It has been reported that one of the mechanisms of COPD in men is the primary testicular dysfunction or the hypo-functioning of the hypothalamic–pituitary–gonadal axis (Van Vliet et al., 2005). Testosterone deficiency also exacerbates COPD symptom through direct impact on respiratory muscle or diminishing exercise capacity (Van Vliet et al., 2005). Thus, clinical use of testosterone replacement therapy (TRT) to treat COPD draws great interest. TRT has been shown to improve body composition, skeletal muscle strength, and exercise capacity in COPD patients (Creutzberg et al., 2003; Svartberg et al., 2004; Caminiti et al., 2009). Clinical data showed a decrease in relative respiratory hospitalization in COPD patients with TRT treatment compared to non-users (Baillargeon et al., 2019), indicating that TRT may slow COPD progression. 

COPD is associated with an abnormal inflammatory response of the airways, the alveoli, and the microvasculature. Cigarette smoke (CS) is the main cause for COPD globally (Rabe and Watz, 2017). The CS-induced inflammatory response in COPD progression involves both innate immunity and adaptive immunity (Pan et al., 2016), which are mediated by multiple immune cell types including macrophages, T lymphocytes, B lymphocytes, and neutrophils, as well as epithelial cells (Retamales et al., 2001). Epithelial cells are activated by CS to produce inflammatory mediators, including tumor necrosis factor α (TNF-α), interleukin-1β (IL-1β), granulocyte‒macrophage colony stimulating factor, IL-8 (Hellermann et al., 2002), and IL-6 (Gan et al., 2004). The nuclear factor-kappa B (NF-κB) pathway is crucial to the pathogenesis of COPD through its transcriptionally regulatory role on cytokines TNF-α, IL-1β, and IL-6 (Edwards et al., 2009). Thus, targeting the NF-κB signaling has been investigated for COPD treatment (Schuliga, 2015). The pharmacological or genetic targeting of NF-κB intermediates, including IKK, has been observed to attenuate inflammation induced by cigarette smoke extract (CSE) or lipopolysaccharide (LPS) (Yang et al., 2009). Inhaled or oral corticosteroids remain the most effective treatment for COPD, which has been proved to effectively inhibit NF-κB activation *in vitro* and *in vivo* (Issa et al., 2007; Salter et al., 2007). However, side effects of long-term corticosteroid treatment should not be ignored, as it affects multiple health systems including immune system, skeletal system, cardiovascular system, integumentary system, muscular system, central nervous system, and gastrointestinal system (Volmer et al., 2018).

Androgen deficiency increases inflammation by upregulating the levels of IL-6, TNF-α, and C-reactive protein (Bobjer et al., 2012; Tsilidis et al., 2013; Tremellen et al., 2018). The anti-inflammatory properties of testosterone have been demonstrated in treating various diseases, including diabetes, autoimmune encephalomyelitis, myasthenia gravis, and coronary artery disease (Duan et al., 2003; Gold and Voskuhl, 2009). Reports have shown that testosterone treatment suppressed TNF-α-induced IL-6 expression in adipocytes and human skin mast cells, suggesting that NF-κB may be involved (Duan et al., 2003; Morooka et al., 2016). Therefore, TRT could be a potential treatment in developing new anti-inflammatory therapeutic strategies for COPD.

Nuclear respiratory factor 1 (NRF1) encodes a transcription factor that regulates the expression of mitochondrial respiratory complex (Bergeron et al., 2001; Piantadosi and Suliman, 2006, 2008), HEME biosynthesis of proteins and DNA transcription, and replication of related factors (Scarpulla, 2002; Kelly and Scarpulla, 2004). Here, we found that NRF1 was upregulated in pulmonary epithelium of COPD model rats and NRF1 transcriptionally regulated p65 expression through chromatin immunoprecipitation (ChIP)‒seq data analysis. Thus, we speculated that NRF1 might be involved in the activation of the NF-κB signaling. The purpose of this study is to assess the function of NRF1 on NF-κB-mediated pulmonary epithelial inflammation in COPD model rats and whether testosterone supplementation executes anti-inflammatory effect through inhibiting NRF1.

## Results

### Testosterone decline is associated with upregulation of NRF1 and p65 in male COPD rats

**Figure 1:**
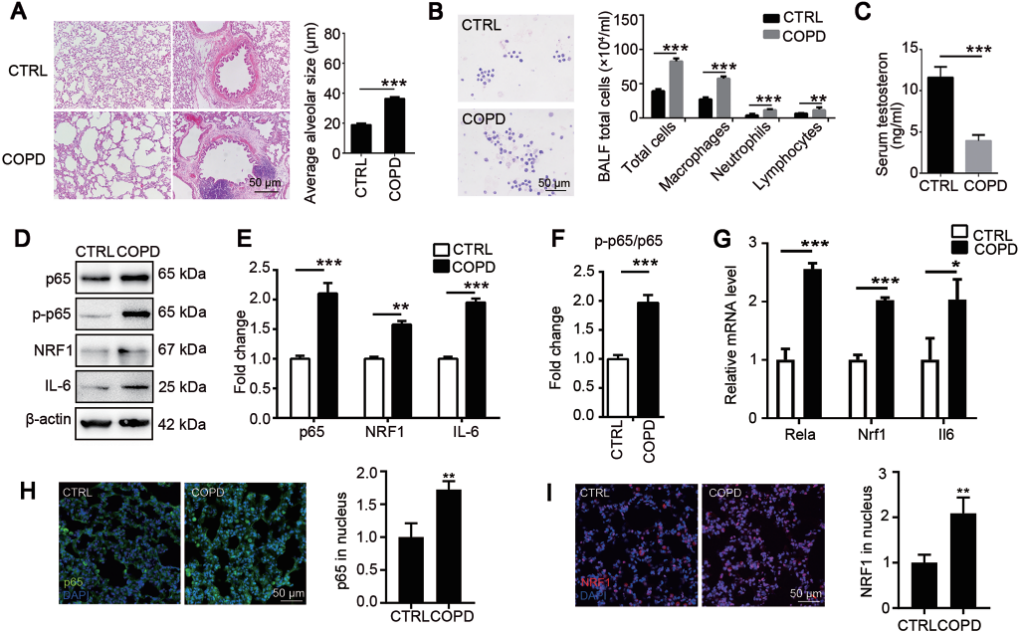
Cigarette‒LPS induces typical pulmonary lesions of COPD. Adult male rats were exposed to CS for 30 min/day for 28 days and 200 μg LPS every 14 days through intratracheal administration. (**A**) H&E staining was used to observe the morphological changes of COPD rats. Average alveolar size was measured and statistically analyzed by *t*-test (200 alveoli per animal were counted). (**B**) Wright staining was used to observe the change of cell number and leukocyte type in BALF. (**C**) Serum testosterone was determined by ELISA. (**D**‒**F**) Pulmonary tissue was lysed for western blotting and relative protein levels were quantified by ImageJ. (**G**) mRNA expression levels of Nrf1, Rela, and Il6 were determined by qRT-PCR. (**H** and **I**) Pulmonary tissues were stained with p65 (**H**) or NRF1 (**I**) antibody for translocation analysis. *n *=* *6, mean ± SEM, *P ≤* *0.05, **P ≤* *0.01, ***P ≤* *0.001 by *t*-test.

To evaluate pulmonary pathology of COPD, we simulated COPD model through co-treatment with LPS and CS in male rats. Irregular large vacuoles as well as inflammatory cell infiltration around the trachea were observed in COPD pulmonary tissue (Figure 1A). More white cells, including macrophages, neutrophils, and lymphocytes, were collected in COPD rat bronchoalveolar lavage fluid (BALF) (Figure 1B), indicating obvious inflammatory response induced in COPD pulmonary tissue. Results in Figure 1D‒G showed that p65 was upregulated and activated along with the proinflammatory cytokine IL-6. In consistence, significant nuclear translocation of p65 in pulmonary tissue of COPD model was observed, further confirming that NF-κB pathway was activated in COPD (Figure 1H). Interestingly, the transcription factor NRF1 was also upregulated (Figure 1D, E, and G) and activated (Figure 1I) in COPD group, proposing that NRF1 might be involved in pulmonary inflammatory response. We also observed a significant reduction of serum testosterone, marking COPD’s influence on gonad function (Figure 1C).

### CSE and LPS synergistically upregulate NRF1 and p65

**Figure 2:**
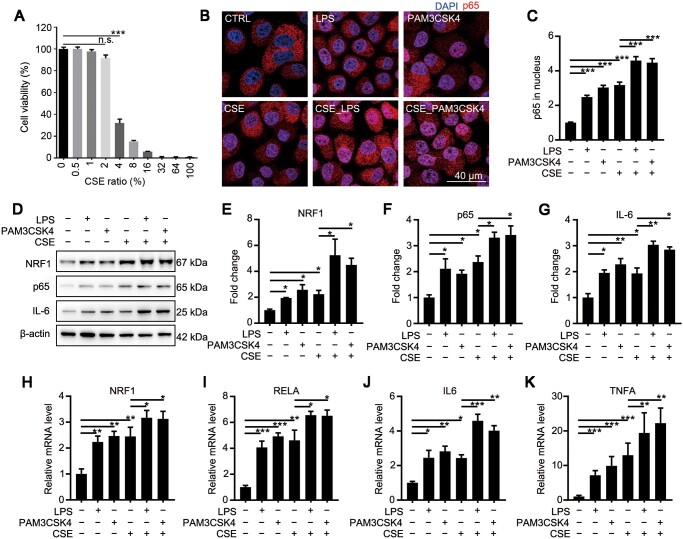
NRF1 and p65 are synchronously upregulated in CSE-, LPS-, PAM3CSK4-, or their combinations-treated L132 cells. (**A**) L132 cells were treated with CSE at indicated concentrations for 48 h. Cell viability was detected through MTT assay. (**B** and **C**) L132 cells were treated with 2% CSE for 42 h, followed by 1 μg/ml LPS or 10 ng/ml PAM3CSK4 for 6 h. Cells were stained with p65 antibody to observe the nuclear translocation and the intensity was quantified. (**D**‒**G**) Protein levels of NRF1, p65, and IL-6 were determined by western blotting. (**H**‒**K**) mRNA expression levels of NRF1, RELA, IL6, and TNFA were determined by qRT-PCR. Mean ± SD, *P ≤* *0.05, **P ≤* *0.01, ***P ≤* *0.001 by one-way ANOVA.

To verify the response of epithelial cells to cigarette stimulation, we diluted CSE to different concentrations and treated L132 cells for 48 h. We tested cell viability through MTT. While <2% CSE showed no significant cytotoxicity, >4% CSE significantly damaged cell viability (Figure 2A). Therefore, we chose 2% CSE as the treating concentration below. To verify whether the activation of p65 was involved in CSE- or LPS-treated cells, L132 cells were exposed to 1 μg/ml LPS for 6 h, 10 ng/ml PAM3CSK4 for 6 h, CSE for 48 h, and the combination of CSE and LPS (CSE_LPS) or CSE and PAM3CSK4 (CSE_PAM3CSK4). Nuclear translocation of p65 was observed in LPS-, PAM3CSK4-, and CSE-treated L132 cells (Figure 2B), indicating that the NF-κB signaling could be activated in pulmonary epithelial cells by CSE. Furthermore, CSE_LPS and CSE_PAM3CSK4 induced more nuclear translocation on p65, suggesting that CSE and LPS synergistically activated the NF-κB signaling (Figure 2C). In consistence, both mRNA and protein levels of p65 and IL-6 were significantly increased after LPS, PAM3CSK4, or CSE treatment and further increased in CSE_LPS or CSE_PAM3CSK4 group (Figure 2D‒K), confirming that the NF-κB signaling stimulated by CSE could be amplified by LPS in pulmonary epithelial cells. Interestingly, the change of NRF1 was synchronous with p65 that was upregulated after LPS, PAM3CSK4, or CSE treatment and further upregulated by the combination of CSE and LPS or PAM3CSK4 (Figure 2D, E, and H), demonstrating that the activation of NRF1 is related to inflammation.

### CSE or LPS induces p65 upregulation through transcriptional regulation by NRF1

**Figure 3:**
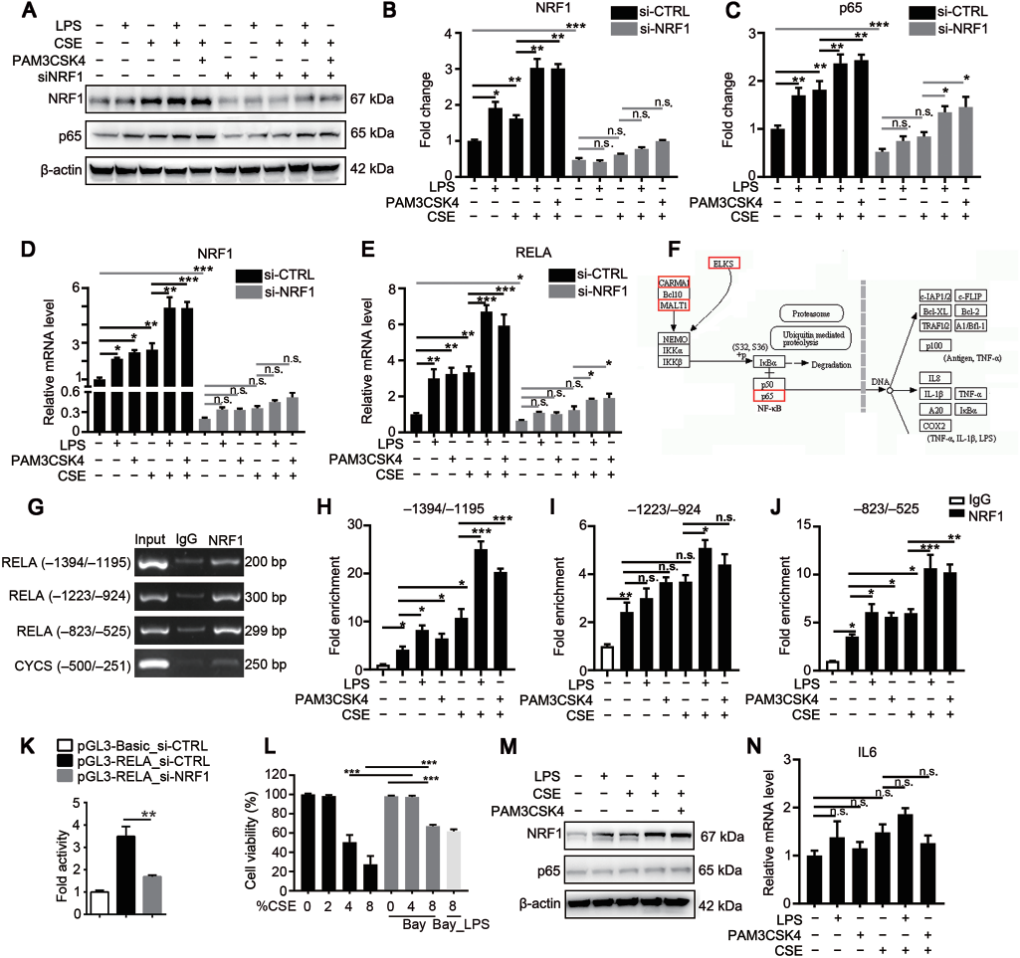
Upregulation of NRF1 transcriptionally activates p65-coding gene RELA in L132 cells exposed to CSE or LPS. (**A**‒**J**) L132 cells were transfected with NRF1 siRNA (si-NRF1) or control siRNA (si-CTRL) for 24 h. Cells were treated with 2% CSE for 42 h followed with 1 μg/ml LPS or 10 ng/ml PAM3CSK4 for 6 h. (**A**‒**C**) Protein levels of NRF1 and p65 were determined by western blotting. (**D** and **E**) mRNA expression levels of NRF1 and RELA were determined by qRT-PCR. (**F**) Enriched DNA fragments by NRF1 were pulled down for ChIP–seq. Target genes were analyzed by MAnorm software. (**G**‒**J**) RELA promoter fragment enrichment of NRF1 was determined by ChIP–qPCR. CYCS, the target gene of NRF1, served as a positive control. (**K**) HEK293T cells were co-transfected with NRF1 siRNA and pGL3-RELA for 48 h. The pRL-TK vector was co-transfected to normalize transfection efficiencies. (**L**‒**N**) L132 cells were pretreated with Bay11-7082 for 24 h and exposed to CSE for 42 h followed with 6-h LPS treatment. Cell viability (**L**) as well as protein levels (**M**) and mRNA expression levels of NRF1 and p65 (**N**) were determined. mean ± SD, n.s. means no significance, *P ≤* *0.05, **P ≤* *0.01, ***P ≤* *0.001 by one-way ANOVA.

To evaluate the relationship between NRF1 and p65, we silenced NRF1 by transfecting NRF1 siRNA (Figure 3A, B, and D). As shown in Figure 3A‒E, silencing NRF1 significantly inhibited p65 expression cells treated with LPS, CSE, PAM3CSK4, or their combinations, suggesting that upregulation of NRF1 in COPD progress is associated with NF-κB signaling activation. It was remarkable that LPS, CSE, PAM3CSK4, and their combinations still partially upregulated the expression of p65, indicating other mechanisms involved in CSE- or LPS-induced inflammation. To further investigate how NRF1 affected p65 expression, we did ChIP–seq analysis to verify the DNA binding site of NRF1 on the promoter region of the p65 gene RELA. As shown in Figure 3F, p65 was a potential NRF1 target gene analyzed by MAnorm software. We further confirmed the binding site of NRF1 through polymerase chain reaction (PCR). Results in Figure 3G showed NRF1 enriched at RELA promoter regions –1394/–1195, –1223/–924, and –823/–525 compared to IgG control. Quantitative real-time PCR (qRT-PCR) results in Figure 3H‒J showed significant increases in the enrichment of RELA promoter regions –1394/–1195 and –823/–525 in L132 cells treated with LPS, PAM3CSK4, CSE, and in particular their combinations, but no significant differences were observed in –1223/–924 enrichment. These results demonstrated that CSE, LPS, and their combinations upregulated the binding of NRF1 to p65 promoter regions, thus rising p65 transcription. To verify the regulation of NRF1 on RELA transcription activity, we co-transfected HEK293T cells with pGL3-RELA, pRL-TK, and NRF1 siRNA. Dual luciferase reporter assay showed downregulated transcription activity of RELA in NRF1-silenced cells, indicating that NRF1 positively regulated RELA transcription (Figure 3K). To further confirm that NRF1 promoted COPD progression through p65, L132 cells were pretreated with 5 μM Bay11-7082 for 24 h to pharmacologically inhibit the NF-κB signaling before CSE and LPS treatments. As shown in Figure 3L, Bay11-7082 pretreatment significantly reduced the cytotoxicity by 4% and 8% CSE. In consistent, p65 along with IL-6 did not respond to LPS, CSE, or combination treatments, while NRF1 was activated (Figure 3M and N), suggesting that NRF1 promoted inflammation by activating the NF-κB signaling.

### Gonadectomy induces COPD deterioration in male instead of female rats

**Figure 4:**
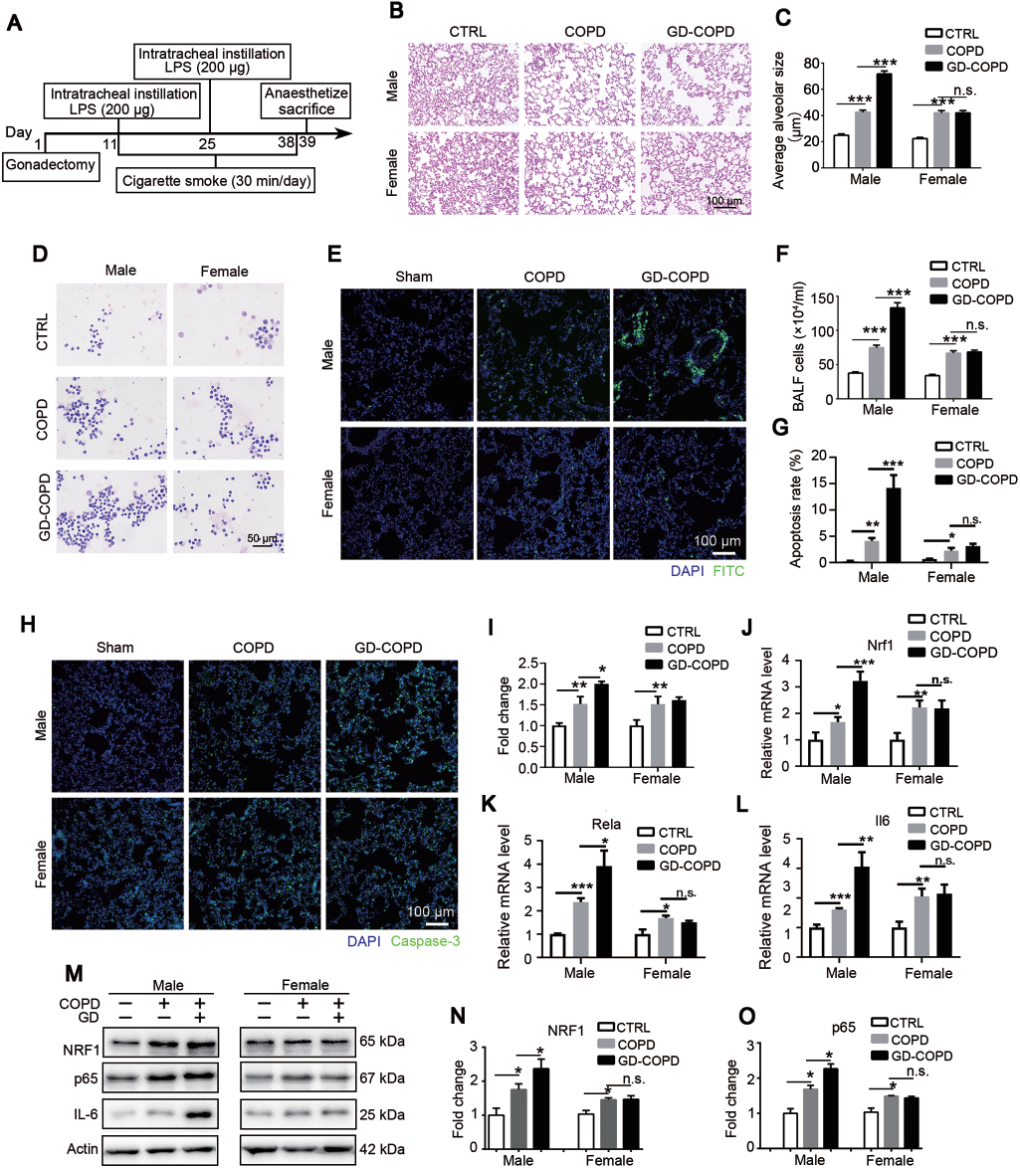
Absence of sex hormones exacerbates pulmonary lesions in male instead of female rats of COPD model. (**A**) Experimental design for the construction of castration in COPD model rats. (**B** and **C**) H&E staining was used to observe the morphological changes of COPD rats. Average alveolar size was measured and statistically analyzed (200 alveoli per animal were counted). (**D** and **F**) Wright staining was used to observe the change of cell number and leukocyte type in BALF. (**E** and **G**) Apoptotic cell nuclei were stained with FITC fluorescence by TUNEL assay. (**H** and **I**) Pulmonary tissues were stained with caspase-3 antibody and intensity of green signal was statistically analyzed. (**J**‒**L**) mRNA expression levels of Nrf1, Rela, and Il6 were measured by qRT-PCR. (**M**‒**O**) Protein levels of NRF1 and p65 were determined by western blotting. *n *=* *6, mean ± SEM, n.s. means no significance, *P ≤* *0.05, **P ≤* *0.01, ***P ≤* *0.001 by one-way ANOVA.

Based on gender difference of COPD, we looked into sex hormone function in COPD progression. A COPD model in gonadectomized rats was established following the instruction in Figure 4A. As shown in Figure 4B and C, no obvious differences of pulmonary morphology were found between male and female COPD rats. However, significant pulmonary deterioration was observed in male gonadectomized rats rather than female ones, suggesting that testosterone played a role in pulmonary protection. In consistent, much more total white cells were collected in male gonadectomized COPD (GD-COPD) rats compared with control or shame COPD rats, but no significant differences were observed in different female groups (Figure 4D and F). We further investigated pulmonary damage by apoptosis induced by LPS/cigarette with terminal deoxyribonucleotidyl transferase-mediated dUTP nick-end labeling (TUNEL) assay and caspase-3 staining. More apoptotic cells were observed in both male and female COPD groups compared with controls, but a significantly higher apoptotic rate was only seen in male rather than female GD-COPD group (Figure 4E and G). Consistently, gonadectomy further upregulated caspase-3 in male instead of female GD-COPD groups (Figure 4H and I). Furthermore, qRT-PCR (Figure 4J‒L) and western blotting (Figure 4M‒O) results showed that NRF1, along with p65 and IL-6, was upregulated in both male and female COPD pulmonary tissues but only further upregulated in male gonadectomized group. All these results proved that absence of testosterone might aggravate the pulmonary inflammation, which involved NRF1-mediated NF-κB signaling.

### Testosterone supplementation alleviates pulmonary lesions of COPD

**Figure 5:**
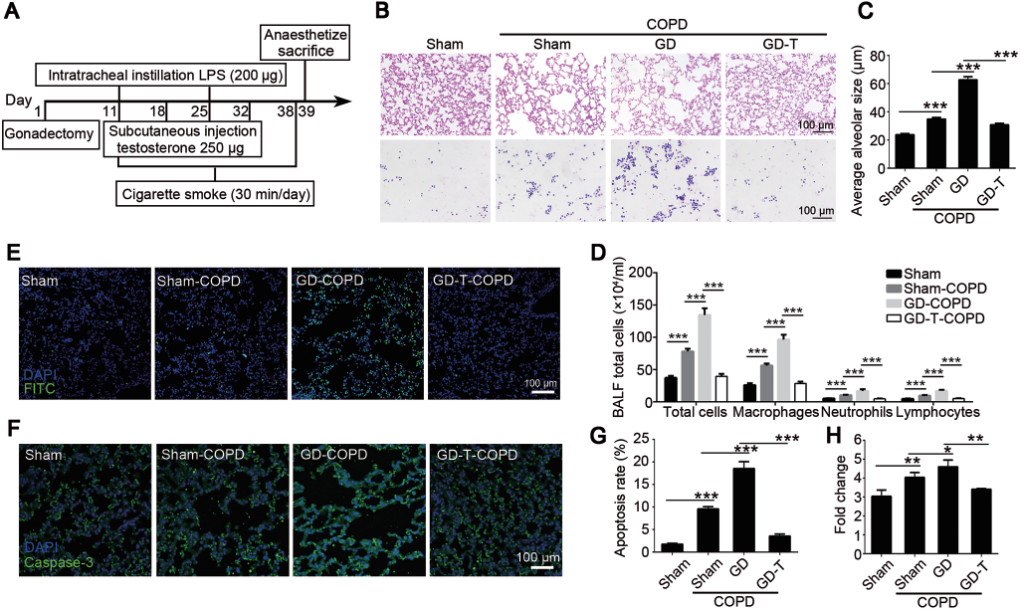
Testosterone supplement alleviates COPD progression in male rates. (**A**) Experimental design for the construction of castration and testosterone supplement model. (**B**‒**D**) H&E staining was used to observe the morphological changes of COPD rats (**B**, top) and average alveolar size was measured and statistically analyzed (**C**, 200 alveoli per animal were counted). Wright staining (**B**, bottom) was used to observe the change of cell number and leukocyte type in BALF (**D**). (**E** and **G**) Apoptotic cell nuclei were stained with FITC fluorescence by TUNEL assay. (**F** and **H**) The tissue was stained with caspase-3 antibody. *n *=* *6, mean ± SEM, *P ≤* *0.05, **P ≤* *0.01, ***P ≤* *0.001 by one-way ANOVA.

To further confirm testosterone function in pulmonary lesions of COPD, male gonadectomized rats with or without testosterone supplementation following COPD stimulation were established as shown in Figure 5A. In consistent with the above results, emphysema was observed in COPD pulmonary tissue and worsened with gonadectomy (GD-COPD). However, testosterone supplementation to GD-COPD (GD-T-COPD) significantly alleviated emphysema (Figure 5B top and C). Similarly, inflammatory cell recruitment, as indicated by white cells collected in BALF, was increased in COPD group, further increased in GD-COPD group, but reduced in GD-T-COPD group (Figure 5B bottom and D). LPS/cigarette-induced pulmonary damage, as indicated by apoptosis with TUNEL assay (Figure 5E and G) and caspase-3 expression (Figure 5F and H), was observed in COPD group and worsened in GD-COPD group but significantly alleviated by testosterone supplementation in GD-T-COPD group. These results suggest that the pulmonary lesions were inversely related to serum testosterone level and testosterone supplementation could alleviate pulmonary lesions in COPD.

### Testosterone supplementation dampens inflammation through NRF1-derived NF-κB signaling in pulmonary tissue of COPD

**Figure 6:**
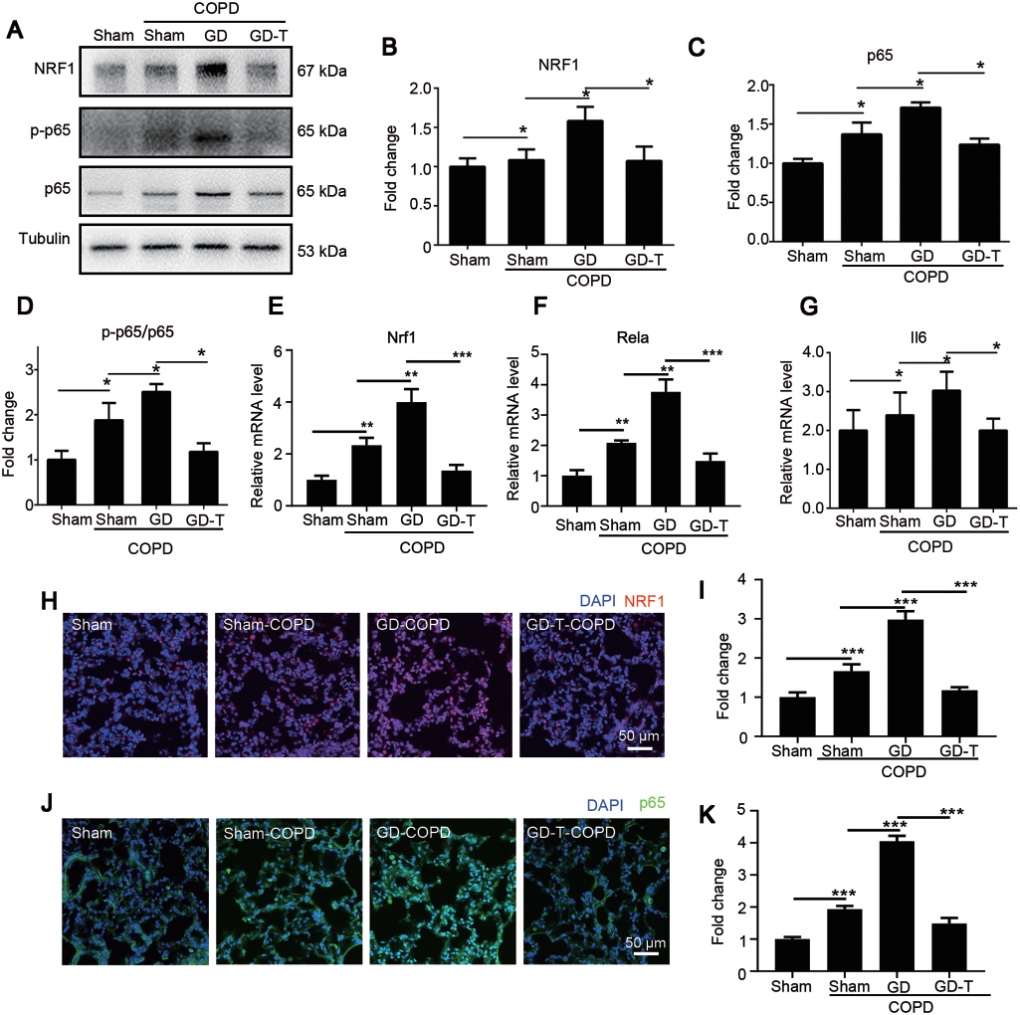
Testosterone supplement restrains pulmonary inflammation of male COPD model. (**A**‒**D**) Protein levels of NRF1, p65, and p-p65 in COPD pulmonary tissues were determined by western blotting and quantified by ImageJ. (**E**‒**G**) mRNA expression levels of Nrf1, Rela, and Il6 were detected by qRT-PCR. (**H**‒**K**) Pulmonary tissues were fixed and stained with NRF1 (**H**) and p65 (**J**) antibodies. Nuclear intensity of NRF1 (**I**) and p65 (**K**) was quantified and statistically analyzed. *n *=* *4, mean ± SEM, *P ≤* *0.05, **P ≤* *0.01, ***P ≤* *0.001 by one-way ANOVA.

To verify whether testosterone affected NRF1-derived NF-κB signaling, we further investigated NRF1 and p65 levels in pulmonary tissue. Both protein (Figure 6A‒C) and mRNA (Figure 6E and F) levels of NRF1 and p65 were significantly upregulated in COPD group and further increased in GD-COPD group but significantly downregulated in GD-T-COPD group compared to GD-COPD group, revealing that testosterone supplementation inhibited NRF1 and p65 expression in COPD. In addition, p65 phosphorylation and the target gene Il6 were significantly upregulated in COPD group and further upregulated in GD-COPD group, while they were downregulated in GD-T-COPD group compared to GD-COPD group (Figure 6D and G), demonstrating that testosterone affected p65 transcription through NRF1 and p65 activation. Further detection for the *in situ* NRF1 and p65 levels by specific antibodies demonstrated that nuclear NRF1 and p65 in pulmonary tissue were increased in COPD group and much higher in GD-COPD group but significantly reduced in GD-T-COPD group (Figure 6H‒K), further confirming that testosterone supplementation inhibited pulmonary inflammation through repressing NRF1 and p65 activation in pulmonary epithelium.

### Testosterone prevents LPS- or CSE-induced inflammation in L132 cells through inhibiting NRF1

**Figure 7:**
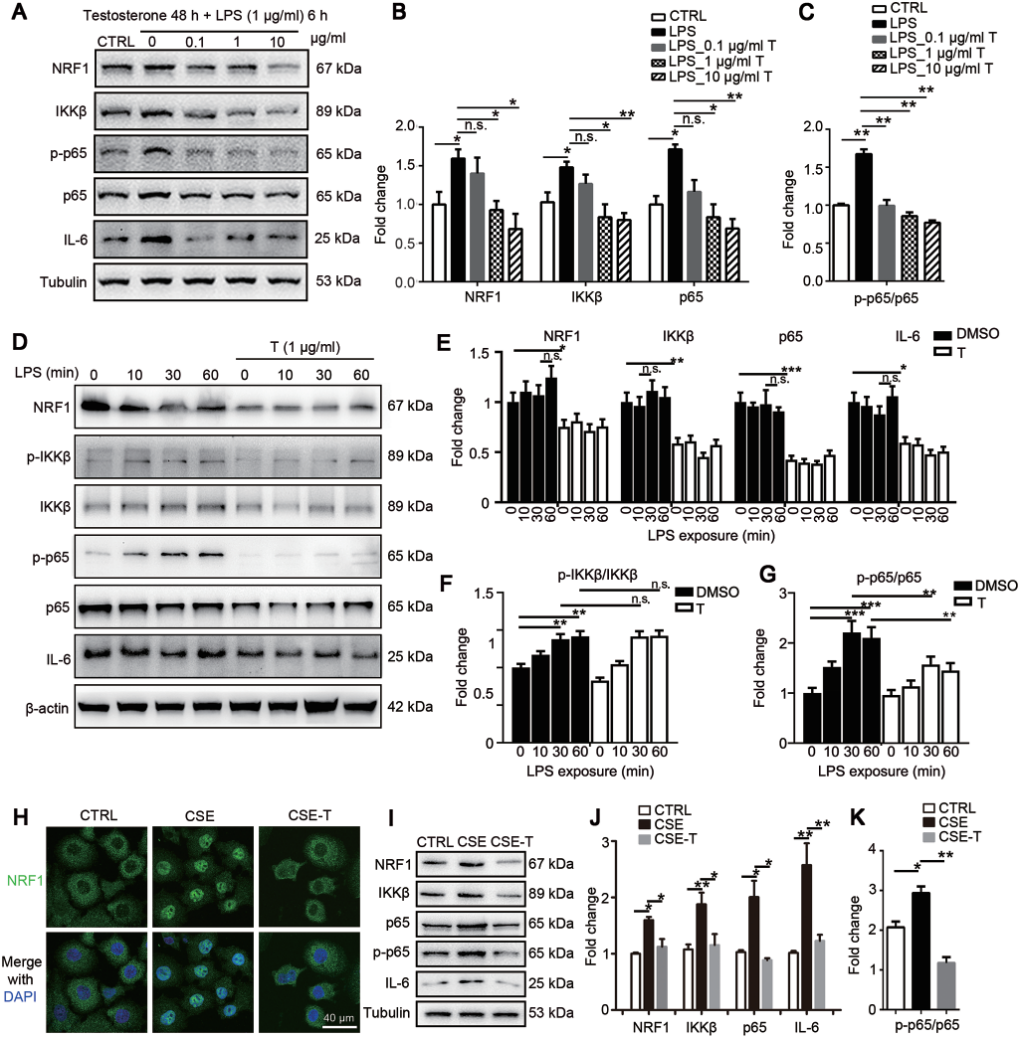
Testosterone dampens inflammatory response in LPS- or CSE-treated L132 cells. (**A**‒**C**) L132 cells were co-treated with LPS and testosterone with indicated concentration and time. Protein levels were determined by western blotting and quantified by ImageJ. Mean ± SEM, *P ≤* *0.05, **P ≤* *0.01 by two-way ANOVA. (**D**‒**G**) L132 cells were pretreated with 1 μg/ml testosterone for 48 h followed by 1 μg/ml LPS for indicated time. Protein levels were determined by western blotting and quantified by ImageJ. Mean ± SEM, *P ≤* *0.05, **P ≤* *0.01, ***P ≤* *0.001 by two-way ANOVA. (**H**‒**K**) L132 cells were co-treated with 2% CSE and 1 μg/ml testosterone for 24 h. (**H**) Cells were stained with NRF1 antibody to observe nuclear translocation. (**I**‒**K**) Protein levels were determined by western blotting and quantified by ImageJ. Mean ± SEM, n.s. means no significance, *P ≤* *0.05, **P ≤* *0.01 by *t*-test.

To verify whether testosterone directly dampens NF-κB-mediated inflammation in pulmonary epithelial cells, we co-treated L132 cells with 1 μg/ml LPS and testosterone at different concentrations. As shown in Figure 7A‒C, testosterone dose-dependently inhibited NRF1, p65, and IL-6, as well as IKKβ and p-p65, suggesting that testosterone prevented LPS-induced inflammation through downregulating NRF1, thus repressing p65 and also repressed p65 phosphorylation through IKKβ. To clarify whether testosterone affected NF-κB signaling activation, cells were pretreated with testosterone for 48 h before treatment with 1 μg/ml LPS for 0, 10, 30, or 60 min. Consistently, testosterone significantly reduced the protein levels of NRF1, IKKβ, p65, and IL-6 (Figure 7D and E). Testosterone pretreatment had no effect on p-IKKβ/IKKβ ratio (Figure 7F), revealing that testosterone did not inhibit IKK activation but significantly inhibited p-p65/p65 ratio (Figure 7G). We speculated that the inhibition of p-p65 was because of the decrease of IKKβ expression. Furthermore, we treated L132 cells with 2% CSE in the presence or absence of 1 μg/ml testosterone to confirm the testosterone function on simulated COPD pulmonary epithelial cells. An obvious nuclear translocation was observed in CSE-treated cells (Figure 7H), confirming that CSE could activate NRF1 in pulmonary epithelial cells. Meanwhile, testosterone supplementation reversed NRF1 nuclear translocation induced by CSE, indicating that testosterone repressed LPS-induced NRF1 activation. In consistent, testosterone supplementation inhibited NRF1, IKKβ, p65, and IL-6 protein levels along with p65 phosphorylation (Figure 7I‒K), confirming that testosterone prevented CSE- or LPS-induced inflammation in L132 cells by repressing the NRF1/NF-κB signaling.

## Discussion

Clinical analysis already showed that TRT has lowered hospitalization and may slow COPD progression (Baillargeon et al., 2019). However, TRT has not been widely used clinically partly because of the unclear efficacy, safety, and side effects. Our work elucidated the function of testosterone in pulmonary inflammatory process of COPD model rats and proved the anti-inflammatory property of testosterone through inhibition of NRF1-derived NF-κB signaling.

CS is the main cause of COPD, and CS-induced COPD animal model has been studied extensively (Jones et al., 2017). In our study, the combination of CS and LPS was used to simulate COPD exacerbation, as demonstrated previously (Nie et al., 2012; Cheng et al., 2019). Model rats showed typical pulmonary bullae, inflammatory symptoms, and a significant increase of NRF1. We further confirmed that NRF1 was involved in inflammation through p65 transcriptional regulation. NRF1 is expected to be upregulated under inflammatory conditions based on its function in mitochondrial biosynthesis. Suliman et al. (2010) reported that NRF1 is upregulated in LPS-exposed mice through transcriptional regulation by NF-κB and CREB, suggesting a complex regulatory network among transcription factors. The proposing mechanism is that the activation of Toll-like receptor 4 induces nuclear translocation of NF-κB, which upregulates NRF1, leading to upregulation of p65 to further exacerbate inflammation. A novel discovery showed that TFAM-dependent mitochondrial synthesis is necessary for NLRP3 inflammasome activation, initiating the maturation and release of IL-1β and IL-18 (Zhong et al., 2018). Li et al. (2018) reported that mtROS and NLRP3 inflammasome take part in ozone-induced lung inflammation. Since NRF1 is a main regulator of TFAM (Thomas et al., 2011) and in the mitochondrial respiratory chain (Scarpulla, 2002), NRF1 might be widely involved in inflammatory response pathways. Because of the synergistic regulation of NF-κB and NRF1, NRF1 might also participate in cytokine storm. Thus, repressing NRF1 may post as a potential treatment for pulmonary inflammation.

We found that suppressing the NF-κB signaling prevented cell toxicity of CSE or LPS. However, 8% CSE still damaged cell viability with or without LPS (Figure 2L), indicating that inflammation was not the only pathological change in CSE-induced COPD. Yoshida et al. (2019) reported that CS-induced ferroptosis is involved in COPD progression. Mizumura et al. (2018) suggested that necroptosis is another critical regulator of cell death in COPD. All these showed complicated pathological changes in CS-induced COPD. Our results in Figures 4 and 5 showed synchronous changes of apoptosis and NF-κB, which were consistent with previous reports (Kasahara et al., 2001; Di Stefano et al., 2002). It is generally believed that NF-κB signaling mediated by TNF is an anti-apoptotic pathway (Liu et al., 2004), but it was also reported that NF-κB promotes Fas-mediated apoptosis (Kuhnel et al., 2000), indicating that NF-κB has dual effects on apoptosis. Moreover, NRF1 is a main regulator of mitochondrial function through transcriptional regulation on CYCS, TFAM, and Complex I to Complex IV, indicating that NRF1 might also participate in apoptosis regulation.

CS‒LPS combined stimulation induced a significant decrease of serum testosterone in male rats, which was also seen in male patients with COPD due to hypoxemia (Atlantis et al., 2013; Wang et al., 2017). We found that male castration increased the recruitment of inflammatory cells and upregulated the expression of TNF-α and IL-6, similar to clinical reports that higher levels of IL-6, IL-1β, and TNF-α were noted in men with testosterone deficiency (hypogonadism) (Maggio et al., 2006; Tsilidis et al., 2013). Testosterone supplementation to physiological level effectively inhibits inflammatory factors and slows down systemic inflammation. The known anti-inflammatory function of testosterone involves repressing Toll-like receptor 4 in macrophages, inhibiting TNF-α-induced mastoparan-1 and IL-6 production in monocytes and mast cells, and affecting T-cell and lymphocyte proliferation (Traish et al., 2018). Here, we provided a mechanism that testosterone suppressing the NF-κB signaling by inhibiting NRF1 expression. We for the first time found a correlation between testosterone and mitochondrial function, indicating that testosterone might be involved in oxidative stress and mitochondrial metabolism. Therefore, testosterone deficiency might not only affect reproductive function and immunity but also play an important role in multiple systems and organs, suggesting that more thorough and well-designed studies are urgently needed to explain the clinical significance of TRT, especially in elderly men with testosterone deficiency.

Estrogen is believed to have a significant anti-inflammatory protective effect, but in our study, we found that pulmonary lesions induced by CS‒LPS were similar between male and female rats, which may provide an explanation that, in recent years, more women suffer from COPD partly due to a rise in female smoking (World Health Organization, 2018). Grabicki et al. (2019) reported that women who smoke less show more exacerbations than men and also a significantly worse prognosis, demonstrating that gender-specific COPD management is necessary. Our study found that only androgen has a protective effect in men, suggesting that female sex hormone replacement therapy may not have therapeutic effect. One important observation that NRF1 was also upregulated in pulmonary tissue of female COPD model rats indicates that targeting NRF1 might be a choice of female COPD treatment.

In summary, the upregulation of NRF1 in COPD pulmonary epithelia transcriptionally stimulates RELA expression, which leads to aggravating pulmonary inflammation. Testosterone deficiency in male COPD rats upregulates NRF1 and accelerates COPD progression. TRT effectively alleviates pulmonary bullae and inflammation. The anti-inflammatory effect of testosterone in pulmonary epithelial cells is through downregulation of NRF1 and inhibition of NF-κB activation.

## Materials and methods

### Animals and treatments

Adult Wistar rats of 140–220 g from the Experimental Animal Center of Nantong University were accommodated for one week before experiments with a 12-h light–dark cycle. The commercial non-filtered cigarettes (Hongtashan) were purchased from China Tobacco Yunnan Industrial Co., Ltd containing 10 mg tar and 1 mg nicotine per cigarette.

Rats were maintained in a 65 cm × 75 cm × 50 cm box with 5 smoldering cigarettes and exposed to CS (total particular matter ∼120 mg/m^3^) for 28 days, 30 min/day. During cigarette exposure, rats were perfused with 200 μg LPS (1 μg/μl) through trachea for every 14 days. To evaluate the sex difference of inflammation in COPD, rats underwent gonadectomy 10 days before COPD construction. Male rats were subcutaneously injected with 1.25 mg/kg b.w. testosterone propionate for every 7 days to supplement testosterone to physiological level. After euthanasia by injection of chloral hydrate, the left pulmonary lobe of rats was fixed and hematoxylin and eosin (H&E) staining was performed. All the studies reported here were submitted to the Ethics Committee on Animal Experimentation of Nantong University, and all procedures were approved according to the Animal Care and Use Committee of Nantong University and the Jiangsu Province Animal Care Ethics Committee (Approval ID: SYXK(SU)2007-0021).

### BALF and Wright stain

Rats were perfused with 3 ml phosphate-buffered saline (PBS) through trachea. Liquids were collected and centrifuged at 400× *g* for 10 min at 4°C. The pellets were resuspended with erythrocyte lysate to remove red blood cells. Cells were suspended in 200 μl PBS and smeared for Wright stain.

### Testosterone determination

Blood was drawn from individual rat’s heart to isolate serum through centrifugation at 2000× *g* for 15 min. Serum was diluted to 20% for testosterone determination through specific ELISA. The test procedure was carried out following the manufacturer’s instructions of the testosterone parameter assay kit (R&D, KGE010). The optical density was determined at 450 and 540 nm wavelengths. Standard curve was created by performing a four-parameter logistic curve fit with Origin 8.0 (https://www.originlab.com).

### TUNEL assay

Rat pulmonary tissues were paraffin-embedded and sectioned to 5 μm. Paraffin sections were dewaxed with xylene and hydrated with gradient alcohol. Then, sections were incubated with proteinase K (20 μg/ml) at 37°C for 30 min. The positive control was treated with DNase I. After reaction with probes, sections were counterstained with DAPI for confocal imaging.

### Preparation of CSE

CSE was prepared by bubbling smoke from one cigarette into 10 ml RPMI 1640 medium without fetal bovine serum (FBS) using an injector in 2 min, according to the method described previously (Yang et al., 2009). CSE solution was adjusted to pH 7.4 and filtered through a 0.22-µm filter. This solution was considered as 100% stock and diluted with culture media for further experiments. The prepared CSE solution was used within 30 min after preparation.

### Cell treatments and toxicity assay

Human pulmonary epithelial cell line L132 was kindly provided by Professor Xiaodong Han (Nanjing University) and cultured in RPMI 1640 medium containing 10% FBS at 5% CO_2_, 37°C. L132 cells were treated with 1 μg/ml LPS or 10 ng/ml PAM3CSK4, a TLR1-2 agonist, for 6 h to activate p65. Cells were treated with 2% CSE for 42 h and co-treated with LPS or PAM3CSK4 for another 6 h to simulate COPD cell model. Cells were seeded at 96-well plates and treated with CSE at different concentrations for 48 h. Cell viabilities were determined by MTT assay.

### Total RNA isolation and qRT-PCR

Total RNA was isolated by TRIzol reagent. Then, 1 μg purified total RNA was reverse-transcribed by HiScript 1st Strand cDNA Synthesis Kit (Vazyme) according to the instructions. qRT-PCR was performed by SYBR premix (Roche). All primers used for qRT-PCR were designed as follows: rat Nrf1, forward 5′-TATGGCGGAAGTAATGAAAGACG-3′, reverse 5′-CAACGTAAGCTCTGCCTTGTT-3′; rat Rela, forward 5′-TTCAACATGGCAGACGACGA-3′, reverse 5′-AGGTATGGGCCATCTGTTGAC-3′; rat Actb, forward 5′-AGATCAAGATCATTGCTCCTCCT-3′, reverse 5′-ACGCAGCTCAGTAACAGTCC-3′; rat Il6, forward 5′-ACAAGTCCGGAGAGGAGACT-3′, reverse 5′-TTCTGACAGTGCATCATCGC-3′. Human NRF1, forward 5′-GTACAAGAGCATGATCCTGGA-3′, reverse 5′-GCTCTTCTGTGCGGACATC-3′; human RELA, forward 5′-ATGTGGAGATCATTGAGCAGC-3′, reverse 5′-CCTGGTCCTGTGTAGCCATT-3′; human 18S rRNA, forward 5′-CAGCCACCCGAGATTGAGCA-3′, reverse 5′-TAGTAGCGACGGGCGGTGTG-3′; human IL6, forward 5′-ACTCACCTCTTCAGAACGAATTG-3′, reverse 5′-CCATCTTTGGAAGGTTCAGGTTG-3′; human TNFA, forward 5′-CACAGTGAAGTGCTGGCAAC-3′, reverse 5′-AGGAAGGCCTAAGGTCCACT-3′; human IL8, forward 5′-TTTTGCCAAGGAGTGCTAAAGA-3′, reverse 5′-AACCCTCTGCACCCAGTTTTC-3′. The relative amount of gene expression normalized to the reference gene was calculated using ΔΔ*C*t, and *C*t means threshold cycle of PCR.

### Western blotting

Pulmonary tissue and cells were lysed with RIPA buffer. Protein concentration was determined by bicinchoninic acid assay. Proteins were isolated by sodium dodecyl sulfate−polyacrylamide gel electrophoresis and transferred to polyvinylidene fluoride membranes using wet trans-blot unit. The membranes were probed with anti-NF-κB p65 (8242, Cell Signaling Technology), anti-phospho-NF-κB p65 (3033S, Cell Signaling Technology), anti-NRF1 (ab175932, Abcam), anti-IL-6 (ab9324, Abcam), anti-IKKβ (8943, Cell Signaling Technology), anti-β-actin (A5316, Sigma), and anti-tubulin (ab7291, Abcam). Binding of primary antibodies was visualized with donkey anti-rabbit HRP-conjugated secondary antibody (305-005-003, Jackson, AB_2339376) or rabbit anti-goat HRP-conjugated secondary antibody (111-005-003, Jackson, AB_2337913) and the ECL Plus system. Grayscale analysis was carried out using ImageJ (National Institutes of Health).

### Immunofluorescence

Sectioned tissue was dewaxed and hydrated. Cultured cells were fixed with 4% paraformaldehyde. Samples were probed with anti-NRF1, anti-NF-κB p65, and anti-cleaved caspase-3 (9661, Cell Signaling Technology) antibodies. Binding of primary antibodies was visualized with Alexa Fluor 488-conjugated anti-rabbit IgG (R37118, Thermo) or Alexa Fluor 555-conjugated anti-goat IgG (A-21432, Thermo), and then samples were counterstained with DAPI. Images were acquired by Leica SP8 confocal microscope with 63× oil immersion objective.

### Dual-luciferase reporter assays

Fragments of human RELA promoter region −1504/−504 were cloned into the *Kpn*I and *Hin*dIII restriction sites of the pGL3-Basic vector (pGL3-RELA). All constructs were verified by sequencing. HEK293T cells (4 × 10^4^) were cultured in 24-well plates in antibiotic-free culture medium the day before transfection. Cells were co-transfected with 500 ng RELA reporter plasmids, 500 ng NRF1 shRNA plasmids, and 10 ng Renilla reporter plasmid (pRL-TK, Promega) as an internal control by Lipofectamine 2000 Reagent (Thermo). Luciferase activities were detected by Dual-Luciferase Reporter Assay System (Promega). Firefly luminescence signal was normalized by Renilla luminescence signal.

### ChIP assay

ChIP experiments were performed using the SimpleChIP^®^ Enzymatic Chromatin IP Kit (Magnetic Beads; Cell Signaling Technology, #9003) according to the manufacturer’s instructions. L132 cells were fixed in culture medium containing 1% formaldehyde at room temperature for 10 min to cross-link proteins and DNA. Then, cells were lysed and incubated with 0.5 μl micrococcal nuclease for 20 min at 37°C to digest DNA to 200–500 bp. The mixture was then immunoprecipitated with 2.5 μg NRF1 antibody (ab34682, Abcam) or a negative control IgG at 4°C overnight. The purified DNA was amplified by qRT-PCR with primers designed as follows: RELA (−1394/−1195) forward 5′-ACAGCCTCAGGAAGCCAAAA-3′, reverse 5′-CCTCGGCGGGGATTTTCC-3′; RELA (−1223/−924) forward 5′-CCAGCGTCTGGGGAAAATC-3′, reverse 5′-CCCTCGCGTGGGAGTT-3′; RELA (−823/−525) forward 5′-CTCCTAACGCTGAGGAAGCC-3′, reverse 5′-CGAGGACGTCAGAGTGGAGA-3′; CYCS forward 5′-TTCCTGTCCGACTGTGGTGT-3′, reverse 5′-GGCGGTCTTGTAGTTCTTGATT-3′.

### Statistical analysis

Data were analyzed with GraphPad Prism v. 6 (GraphPad) by *t*-test, one-way ANOVA, or two-way ANOVA followed by Tukey’s test. All the data were presented as mean ± SEM or mean ± SD. *P ≤* *0.05, **P ≤* *0.01, ***P ≤* *0.001.
